# Spider Venom-Derived Peptide Exhibits Dual Anti-Inflammatory and Antioxidative Activities in LPS-Stimulated BEAS-2B Cells

**DOI:** 10.3390/antiox14121485

**Published:** 2025-12-11

**Authors:** Jin Wook Oh, Min Kyoung Shin, Hye-Ran Park, Sukin Jeong, Minho Lee, Ji Hyuk Ko, Jae Young Lee, Seung-Cheol Jee, Jung-Suk Sung

**Affiliations:** Department of Life Science, Dongguk University-Seoul, Goyang 10326, Republic of Korea; oh5929@dongguk.edu (J.W.O.); shinmk94@dgu.ac.kr (M.K.S.); 2019111678@dongguk.edu (H.-R.P.); jeong9414@dgu.ac.kr (S.J.); minholee@dgu.edu (M.L.); komes007@dongguk.edu (J.H.K.); jylee001@dongguk.edu (J.Y.L.); markjee@dongguk.edu (S.-C.J.)

**Keywords:** respiratory tract damage, anti-inflammation, antioxidant, therapeutic peptide

## Abstract

Most respiratory diseases are driven by excessive airway inflammation and oxidative stress, yet current therapies often lack durable efficacy or are unsafe. Host-defense peptides, commonly enriched in animal venoms, offer diverse, target-selective scaffolds for new therapeutics. In this study, we aimed to discover a novel bioactive peptide with therapeutic potential on respiratory tract damage by utilizing *Nephila clavata* venom gland transcriptome. Using in silico analysis and machine learning-based functional prediction, we designed a peptide, NC-CV, expected to have dual anti-inflammatory and antioxidant activities with low cytotoxicity. In experimental validation, NC-CV improved human bronchial epithelial BEAS-2B cell viability under lipopolysaccharide (LPS) exposure while reducing LPS-induced pro-inflammatory cytokine expression and intracellular reactive oxygen species (ROS) generation. Mechanistic studies and molecular docking simulations indicated that NC-CV prevents toll-like receptor 4 signaling activation, suppressing nuclear factor κB and mitogen-activated protein kinase pathways. Moreover, the antioxidant activity of NC-CV was primarily based on direct intracellular ROS scavenging rather than the induction of endogenous antioxidant enzymes. Collectively, these findings demonstrated that the venom-derived peptide NC-CV disrupts the self-reinforcing cycle involving inflammatory signaling and oxidative stress in airway epithelium, highlighting its promise as a therapeutic candidate for respiratory disease.

## 1. Introduction

Respiratory diseases represent a major global health burden, encompassing a wide range of conditions, from acute infections to chronic inflammatory disorders like acute respiratory distress syndrome, asthma, and chronic obstructive pulmonary disease [1,2,3]. These pathologies share a pathological feature, progressive airway epithelial damage, which disrupts the primary barrier against inhaled pathogens, allergens, and toxins [4]. Once triggered, the sustained inflammation and impaired mucociliary clearance associated with this damage ultimately compromise respiratory function [5]. Current therapeutic approaches addressing these conditions are hampered by significant adverse effects, including immunosuppression and steroid resistance [6]. Moreover, they often only address a subset of symptoms, rather than simultaneously targeting the interconnected mechanisms that drive the disease’s pathology.

Lipopolysaccharide (LPS), which comprises the outer membrane of Gram-negative bacteria, is one of the most prevalent inducers of inflammatory responses in the airway epithelium [7,8]. Upon LPS exposure, epithelial cells initiate innate immune recognition through the toll-like receptor 4 (TLR4) pathway, in which LPS binds to myeloid differentiation factor 2 (MD2), forming a complex that facilitates TLR4 dimerization and activation [9,10]. This triggers the recruitment of adaptor proteins, notably myeloid differentiation primary response 88 (MyD88), initiating a cascade that activates mitogen-activated protein kinase (MAPK) family proteins—including extracellular signal-regulated kinase (ERK), c-Jun N-terminal kinase (JNK), and p38—and the nuclear factor κB (NFκB) pathway [11]. The phosphorylation and subsequent nuclear translocation of NFκB, along with MAPK-dependent transcription factor activation, culminate in the robust upregulation of pro-inflammatory mediators, including interleukin-1 beta (IL-1β), tumor necrosis factor-alpha (TNF-α), and various chemokines [12]. The unregulated upregulation of these mediators amplifies local inflammation and increases vascular permeability, establishing the self-perpetuating cycle of tissue injury characteristic of many respiratory diseases.

Beyond inflammatory signaling, LPS induces profound oxidative stress in airway epithelial cells, creating a pathogenic synergy that exacerbates tissue damage [13]. In this process, TLR4 activation stimulates NADPH oxidase (NOX) enzymes, which generate superoxide anions and reactive oxygen species (ROS), while mitochondrial dysfunction further contributes to ROS accumulation [14]. When ROS production exceeds cellular antioxidant capacity, it can cause lipid peroxidation, DNA damage, and, ultimately, cell death. Critically, inflammation and oxidative stress engage in a bidirectional crosstalk that creates a vicious cycle: ROS activate MAPK/NFκB signaling, while inflammatory cytokines upregulate ROS-generating enzymes. In chronic respiratory diseases, this sustained inflammatory–oxidative axis drives an irreversible airway remodeling process that leads to progressive lung function loss [15].

Bioactive peptides have emerged as promising therapeutic agents due to the diversity of their biological activities and their target specificity [16,17]. Comprised of short amino acid sequences, these exhibit antimicrobial, anti-inflammatory, antioxidant, and/or immunomodulatory properties and act through mechanisms distinct from conventional drugs [18,19,20]. Host-defense peptides (HDPs) represent a particularly potent bioactive reservoir, functioning as innate immunity components that combat pathogens while also modulating host homeostasis [21]. Animal venoms are exceptionally rich sources of HDPs, as venom components have evolved to be suitable for hunting and defense [22]. Among them, spider venoms contain especially vast peptide repertoires, with 50,000 species producing unique profiles shaped by distinct environmental niches. Advances in transcriptomics now enable the systematic exploration of these libraries, facilitating the identification of novel therapeutic candidates through in silico prediction and analysis.

In this study, we analyzed the transcriptome of *Nephila clavata*’s spider venom glands and, through an in silico screening based on HDP structural features, we identified an 18-mer peptide NC-CV with dual anti-inflammatory and antioxidative activities for use in respiratory disease treatments. Functional characterization in LPS-stimulated BEAS-2B cells demonstrated that NC-CV attenuates inflammatory responses, reduces intracellular ROS, and improves cell viability, and mechanistic investigations revealed that NC-CV competitively binds MD-2 in the TLR4–MD-2–LPS complex, disrupting LPS recognition and receptor dimerization, while also exhibiting potent NRF2-independent antioxidant activity through direct radical scavenging. These findings establish NC-CV as a bifunctional therapeutic candidate capable of simultaneously disrupting the inflammatory–oxidative stress cycle that drives respiratory disease pathogenesis.

## 2. Materials and Methods

### 2.1. Sample Preparation and Transcriptome Analysis

Venom glands were isolated from male and female *Nephila clavata*, and the total RNA was extracted using the RNeasy Mini Kit (Qiagen, Hilden, Germany) according to the manufacturer’s instructions, and RNA sequencing was performed by Macrogen (Seoul, Republic of Korea). The raw RNA-seq reads were first evaluated with FastQC v0.12.1, and adapter/low-quality sequences were trimmed using Trimmomatic v0.36. Reads shorter than 36 bp after trimming were discarded [23,24].

De novo transcriptome assembly was conducted using Trinity v2.15.2 with strand-specific paired-end reads [25]. The assembly quality was evaluated using the total number of transcripts, N50, and average length statistics, and assembly completeness was assessed using BUSCO v5.6.1 based on the Arthropoda dataset. The BUSCO analysis identified 99.0% of the genes as complete (51.9% single-copy and 47.1% duplicated), with only 0.4% fragmented and 0.6% missing, out of 1013 expected orthologs, indicating a highly complete assembly [26]. After removing transcripts shorter than 200 bp, the final filtered assembly was used as the reference for downstream analyses.

Transcript abundance was estimated with Salmon v1.10.0 in quasi-mapping mode, using a Trinity-based index. Genes with <1 count per million in at least two samples were filtered out [27]. A differential expression analysis was then performed in R v4.5.1 using the *edgeR* package (Bioconductor, v3.42.4), employing the TMM method for normalization and the negative binomial model for statistical testing (Table S1) [28]. Sex (female vs. male) was included as the main factor, with three biological replicates per group. Significance was determined at FDR < 0.05 and |log_2_FC| ≥ 1, with *p*-values adjusted using the Benjamini–Hochberg procedure.

### 2.2. Protein Prediction, Annotation, and Toxin Candidate Identification

Open reading frames were predicted from assembled transcripts using TransDecoder v5.7.1, and translated protein sequences were annotated using BLASTp v2.2.31 searches against the UniProtKB and the UniProtKB ToxProt (Animal Toxin Annotation Project) dataset (Table S2) [29]. Significant hits were defined at an *E*-value ≤ 1 × 10^−5^, given appropriate coverage and identity thresholds. Functional annotations were further refined by comparison to curated toxin/venom databases.

Putative secretory proteins were identified using SignalP v5.0, employing the default parameters for eukaryotes [30]. Only sequences predicted to contain N-terminal signal peptides were considered as candidate secreted venom components. When relevant, sequences were cross-checked for conserved toxin domains and signal peptide predictions to avoid false positives (Table S1).

### 2.3. In Silico-Based Therapeutic Peptide Predictions and Peptide Synthesis

The venom gland transcriptome of *Nephila clavata* was analyzed to discover novel therapeutic peptide sequences. Toxin and venom protein sequences from the UniProt database were utilized for homology analysis. For each peptide, the physicochemical properties and three-dimensional structural model were assessed using Protein Calculator (https://pepcalc.com/protein-calculator.php, accessed on 15 January 2025), XtalPred (https://xtalpred.godziklab.org/XtalPred-cgi/xtal.pl, accessed on 15 January 2025), Pepfold4 (https://bioserv.rpbs.univ-paris-diderot.fr/services/PEP-FOLD4/, accessed on 16 January 2025), and HeliQuest version 2 [31,32,33]. Six web tools were employed to predict the anti-inflammatory, antioxidant, and hemolytic activities of the peptides: PepNet (http://liulab.top/PepNet/server, accessed on 5 February 2025), PreAIP (http://kurata14.bio.kyutech.ac.jp/PreAIP/, accessed on 5 February 2025), AIPID version 1, AnOxPpred-1.0, Peptide.Bio v0.19.0, and the Database of Antimicrobial Activity and Structure of Peptides v3 (DBAASP) [34,35,36,37,38]. After the in silico analysis, selected peptides were synthesized by Biostem (Ansan, Republic of Korea). Peptides with over 95% purity, confirmed by HPLC and mass spectrometry, were dissolved in distilled water, and aliquots were then stored at −80 °C until further use (Figure S1).

### 2.4. Cell Culturing

Human bronchial epithelial cells (BEAS-2B; ATCC CRL-3588) and THP-1 monocytes (ATCC TIB-202) were purchased from the American Type Culture Collection (ATCC; Manassas, VA, USA) and cultured in RPMI 1640 medium (Welgene Inc., Gyeongsan, Gyeongsangbuk-do, Republic of Korea) containing fetal bovine serum (FBS; Gibco, Grand Island, NY, USA) at 10%, penicillin/streptomycin (Welgene) at 1%, and sodium pyruvate (Welgene) at 1%. HDFα (ATCC PCS-201-012) cell lines were cultured in high glucose DMEM (Welgene) containing FBS (Gibco) at 10% and penicillin/streptomycin (Welgene) at 1%. All cell lines were cultured at 5% CO_2_ and 37 °C in a humid incubator.

### 2.5. Cell Viability Assay

To assess the effect of NC-CV on cell viability, a cell viability assay was performed using Quanti-Max WST-8 Cell Viability Assay Solution (Bio-max, Seoul, Republic of Korea). Cells were seeded at 1 × 10^4^ cells per well in 96-well plates and incubated with varying peptide concentrations for 24 h. After peptide treatment, 100 μL of a 10:1 culture medium–WST-8 solution mixture was added to each well. The plates were then incubated at 37 °C for 30 min, and the absorbance at 450 nm was measured using a microplate reader (Molecular Devices, San Jose, CA, USA). Cell viability was calculated as a percentage relative to the control.

### 2.6. Reverse Transcription-Quantitative Polymerase Chain Reaction (RT-qPCR) Analysis

The total mRNA was extracted from BEAS-2B cells using TRIzol Reagent (Invitrogen, Thermo Fisher Scientific, Waltham, MA, USA), and the RNA concentration was measured using a NanoDrop 2000 spectrophotometer (Thermo Fisher Scientific). Subsequently, 2000 ng of total mRNA was reverse-transcribed into cDNA using M-MLV Reverse Transcriptase (ELPISBIO, Daejeon, Republic of Korea). The RT-qPCR analyses were conducted in a 20 μL reaction mixture containing 10 μL of SYBR Green PCR Master Mix, 1 μL each of the forward and reverse primers (Table 1), 1 μL of cDNA template, and 7 μL of distilled water. Amplification was performed using a CFX Connect Real-Time PCR Detection System (Bio-Rad, Hercules, CA, USA), with the thermal cycling conditions including an initial denaturation step at 95 °C for 3 min, followed by 45 cycles of denaturation at 95 °C for 10 s, annealing at 60 °C for 30 s, and extension at 72 °C for 30 s.

### 2.7. Intracellular Reactive Oxygen Species Detection Assay

Intracellular ROS production was assessed using the fluorescent probe 2′,7′-dichlorodihydrofluorescein diacetate (DCFH-DA; Sigma-Aldrich, St. Louis, MO, USA). Following LPS stimulation with or without a peptide treatment for 24 h, cells were washed with Dulbecco’s phosphate-buffered saline (DPBS; Biosolution, Seoul, Republic of Korea) and incubated in 10 μM DCFH-DA in DPBS at 37 °C for 30 min in darkness. Fluorescence intensity was then measured using an Infinite F200 Pro Multimode Microplate Reader (Tecan, Männedorf, Switzerland) at excitation and emission wavelengths of 485 and 535 nm, respectively.

### 2.8. Immunocytochemistry Staining

For immunocytochemistry staining, glass coverslips pre-coated with poly-L-lysine (Sigma-Aldrich) were placed in 6-well plates and sterilized with 70% ethanol and ultraviolet light. Cells (2.5 × 10^5^ cells/well) were seeded on the coverslips and then LPS with or without NC-CV was applied, and the plates were incubated for 24 h. The samples were then fixed in 4% formaldehyde (Sigma-Aldrich) for 15 min and permeabilized with 0.25% Triton X-100 (Sigma-Aldrich) for 10 min. After blocking with 1% bovine serum albumin (Sigma-Aldrich) in 1× TBST (Sigma-Aldrich) for 45 min, the samples were incubated with primary antibodies for 1 h. The samples were then washed three times with cold TBST and incubated with the secondary antibodies for another 45 min. After an additional four washes with TBST, nuclei were stained with 1 μg/mL 4′,6-diamidino-2-phenylindole for 1 min. The coverslips were then mounted onto glass slides, and fluorescent images were acquired using a confocal laser scanning microscope (Carl Zeiss, Oberkochen, Germany).

### 2.9. Western Blotting Analysis

Cells were lysed in RIPA buffer (Biosolution) supplemented with protease and phosphatase inhibitor cocktails (Sigma-Aldrich), and protein concentrations were determined using the Pierce™ BCA Protein Assay Kit (Thermo Fisher Scientific) according to the manufacturer’s protocol. Equal amounts of protein were separated using 10% sodium dodecyl sulfate-polyacrylamide gel electrophoresis and transferred onto polyvinylidene difluoride membranes (GE Healthcare, Chicago, IL, USA). The membranes were blocked with 5% skim milk (Difco Laboratories, Franklin Lakes, NJ, USA) in 1× TBST for 1 h, and then incubated at 4 °C in a shaker with primary antibodies overnight. After four washes with 1× TBST, the membranes were incubated for 45 min at room temperature with horseradish peroxidase-conjugated secondary antibodies. All antibodies were applied as 1:1000–1:2000 dilutions in 1% skim milk solution. After additional washings, signals were visualized using ECL Plus Western blotting detection reagents (Amersham Bioscience, Buckinghamshire, UK) in a ChemiDoc™ Imaging System (Bio-Rad Laboratories, Hercules, CA, USA). Band intensities were quantified using Image Lab™ Software version 4.0.1 (Bio-Rad).

### 2.10. Radical Scavenging Assay

The radical scavenging activities of the peptides were evaluated using 2,2-diphenyl-1-picrylhydrazyl (DPPH) assays. Peptides were serially diluted in distilled water, and then 100 μL of peptide or vehicle control was mixed with an equal volume of 0.2 mM DPPH in 80% ethanol in a 96-well plate. A mixture of distilled water and 80% ethanol served as a blank for quantification. Plates were incubated for 30 min at room temperature in the dark with shaking, and absorbance at 570 nm was then measured using a microplate reader (Molecular Devices).

### 2.11. Ferrous Ion Chelating (FIC) Assay

The metal chelating activities of the peptides were evaluated using the AOX-15 Ferrous Ion Chelating Assay Kit (Zenbio, Inc., Durham, NC, USA) according to the manufacturer’s instructions. Fifty μL of FeSO_4_ working solution, 50 μL of the peptide sample, and 100 μL of ferrozine solution were mixed, and the mixtures were then incubated for 10 min at room temperature in the dark. Absorbance at 570 nm was measured using a microplate reader (Molecular Devices).

### 2.12. Molecular Docking Simulation

The crystal structure of the TLR4–MD-2–LPS complex (PDB ID: 3FXI) was obtained from the Protein Data Bank (PDB) and employed as the receptor for molecular docking analyses [39]. The LPS-binding site on MD-2, the TLR4–MD-2 interaction sites, and the dimerization interface of the TLR4–MD-2–LPS complex were mapped, and a corresponding grid box was defined [10,39]. The TLR4 and MD-2 models were prepared for individual molecular docking simulations using AutoDockTools version 4.2 (ADT) [40]. This preparation involved the separation of individual chains, removal of co-crystallized ligands, addition of polar hydrogens, and assignment of side-chain flexibility to binding site residues, while the main-chain atoms forming α-helices were kept rigid. Finally, the TLR4 and MD-2 structures were converted from the PDB format to the PDBQT format.

The three-dimensional structure of NC-CV was generated using ColabFold (version 1.5.5) [41]. The predicted model was evaluated based on predicted local distance difference test (pLDDT) scores, with high-confidence regions (pLDDT > 70) considered to be reliable for subsequent molecular docking studies. Polar hydrogens were added, side-chain flexibility was assigned to residues, and the file format was converted from PDB to PDBQT using ADT [40]. Molecular docking simulations and scoring for the processed TLR4, MD-2, and NC-CV structures were performed using AutoDock Vina version Vina 1.2.0 [42].

The molecular docking results were scored using the “Protein Interfaces, Surfaces and Assemblies” service at the European Bioinformatics Institute (http://www.ebi.ac.uk/pdbe/prot_int/pistart.html, accessed on 30 September 2025) website, while non-covalent and hydrogen-bond interactions were analyzed using PDBsum (https://www.ebi.ac.uk/thornton-srv/databases/pdbsum/Generate.html, accessed on 2 October 2025) and Discovery Studio Visualizer (BIOVIA, Dassault Systèmes, San Diego, CA, USA, 2025) (Table S3). All results were visualized with PyMOL (The PyMOL Molecular Graphics System, Version 3.1 Schrödinger, LLC, New York, NY, USA) [43].

### 2.13. Statistical Analysis

All experiments were performed in triplicate, and data are presented as means ± the standard error of the mean (SEM). Statistical analyses were conducted using one-way ANOVAs followed by Tukey’s post hoc tests in GraphPad Prism 5.0 (GraphPad Software, La Jolla, CA, USA). For all tests, *p*-values below 0.05 were considered statistically significant.

## 3. Results

### 3.1. A Novel Functional Peptide from the N. clavata Venom Gland Transcriptome

**Figure 1:**
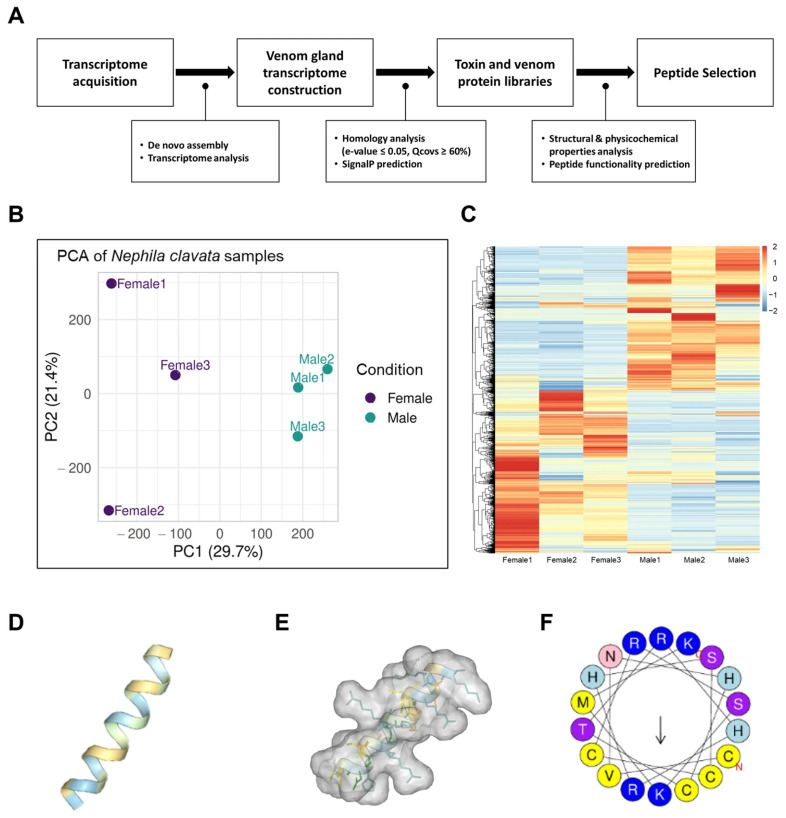
(**A**) Workflow used in this study for peptide discovery and the identification of NC-CV from *N. clavata* spider venom gland transcriptomes. (**B**) A principal component analysis plot and (**C**) a hierarchical clustering tree with the associated heatmap of assembled transcriptome data from female and male spiders. The α-helical propensity and three-dimensional structures of candidate peptides were predicted using (**D**,**E**) PEP-FOLD4 and (**F**) HeliQuest, respectively.

In search of functional peptides, we examined transcriptome data from the venom gland of the spider *N. clavata* (Figure 1A). Given the ecological differences between spider sexes, sex-specific biological features, including venom components, are common [44,45]. Thus, to increase the total available data for bioactive peptide identification, we obtained venom gland transcriptomes from both male and female spiders. The raw transcriptome data from RNA-sequencing were subjected to de novo analysis, protein prediction, annotation, and, ultimately, toxin candidate identification.

Expression profiles were first analyzed based on the assembled transcriptome. A principal component analysis (PCA) revealed a clear separation between sexes, with replicates clustering tightly within the groups, indicating high reproducibility and distinct sex-specific global expression patterns (Figure 1B). Consistent patterns were confirmed by hierarchical clustering and heatmap visualization, which again produced coherent within-group profiles and sharp contrasts between female and male samples (Figure 1C).

Next, homology analysis was conducted using the constructed venom gland transcriptome libraries to identify venomous protein components. Based on the curated Animal Toxin Annotation Project (ToxProt) database from UniProtKB, the analysis identified transcripts with high similarities to known toxin and venom proteins. Strict quality and relevance criteria were applied to ensure that candidate toxin and venom transcripts were both valid and functionally meaningful: structural homology to UniProtKB ToxProt entries, requiring an *E*-value ≤ 0.05 and query coverage ≥ 60%, provided statistical confidence in annotations; the presence of signal peptides confirmed the transcripts’ secretory potential; and a high expression threshold, ≥ 40 TPM (transcripts per million), ensured physiological relevance. This process yielded 37 toxin and venom protein candidate libraries.

Among candidate sequences, structural and physicochemical properties were investigated via in silico analyses. Since an α-helical secondary structure is a representative characteristic of HDPs, five α-helix-enriched transcripts were selected. Their helical regions were then segmented into a series of 18-mer peptides, resulting in 26 final candidates exhibiting physicochemical features characteristic of HDPs. The anti-inflammatory activities, antioxidant capacities, and cytotoxicities of all 26 candidate peptides were predicted using machine learning-based tools, followed by an overall scoring of the predictions. An 18-mer sequence derived from transcript DN214 was calculated to have a net positive charge of +5 and an α-helical structure (Figure 1D,E), and it was predicted to be an anti-inflammatory peptide (AIP) and antioxidative peptide (AOP) with low cytotoxicity (Table 2 and Table 3). Also, the peptide was enriched in cysteine and histidine residues, qualities associated with enhanced radical scavenging activity (Figure 1E). The sequence was designated as NC-CV and synthesized for functional evaluation.

### 3.2. NC-CV Attenuates LPS-Induced Inflammation and ROS Production in BEAS-2B Cells

**Figure 2:**
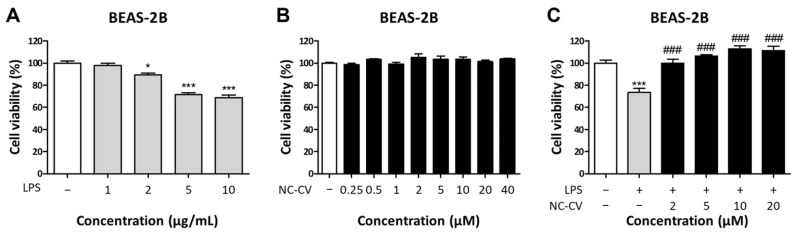
NC-CV reduces LPS-induced cell death in BEAS-2B cells. The BEAS-2B cells were treated for 24 h with (**A**) LPS alone and (**B**) NC-CV alone at the indicated concentrations. (**C**) In cells stimulated with LPS at 10 μg/mL, the protective effects of 2, 5, 10, and 20 μM NC-CV co-treatments on cell viability were assessed. Cell viability was measured using the WST-8 assay. Statistical significance is denoted using symbols: * (*p* < 0.05) and *** (*p* < 0.001) for comparisons between the treatment and control groups and ### (*p* < 0.001) for comparisons between the NC-CV-treated and LPS only treated groups.

To evaluate the anti-inflammatory and antioxidant effects of NC-CV, cytotoxicity assays were performed on bronchial epithelial cell line BEAS-2B cells treated with LPS and/or NC-CV. The BEAS-2B cells were exposed to LPS at concentrations ranging from 1 to 10 μg/mL to determine the optimal concentration for inducing cellular damage, and 10 μg/mL was selected for use in further experiments, as it induced the greatest reduction in cell viability (Figure 2A). Alone, NC-CV showed no significant cytotoxicity in BEAS-2B cells across concentrations ranging from 0.25 to 40 μM, and this non-cytotoxicity was consistent in the other tested cell lines (Figure 2B and Figure S2); therefore, concentrations of 2, 5, 10, and 20 μM were selected for subsequent functional studies. Under LPS stimulation at 10 μg/mL, co-treatment with NC-CV improved the cell viability of BEAS-2B cells in a dose-dependent manner (Figure 2C).

**Figure 3:**
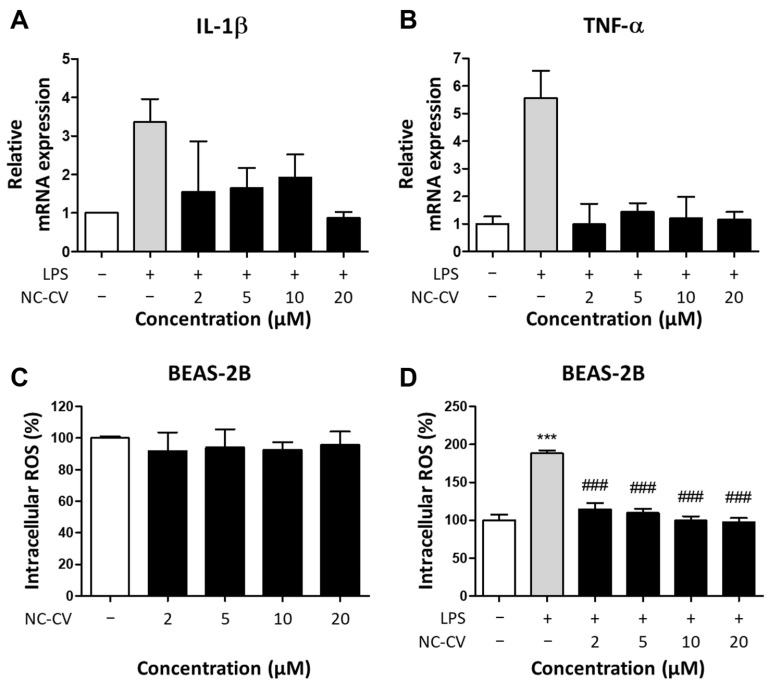
NC-CV suppresses LPS-induced pro-inflammatory cytokine expression and intracellular ROS production in BEAS-2B cells. In all experiments, BEAS-2B cells were treated with 10 μg/mL of LPS and/or 2, 5, 10, 20 μM NC-CV for 24 h. (**A**,**B**) The mRNA expression levels of IL-1β and TNF-α were analyzed using RT-qPCR and normalized to the expression of housekeeping genes. Data are presented relative to the expression levels in the untreated control cells. (**C**,**D**) Intracellular ROS levels were measured using DCF-DA fluorescence after treatment with (**C**) NC-CV alone or (**D**) LPS with or without NC-CV co-treatments. Statistical significance is denoted using symbols: *** (*p* < 0.001) for comparisons between the treatment and control groups and ### (*p* < 0.001) for comparisons between the NC-CV-treated and LPS only treated groups.

Next, the mRNA levels of pro-inflammatory cytokines IL-1β and TNF-α were quantified by RT-qPCR to determine whether NC-CV attenuates the LPS-induced inflammatory response. In BEAS-2B cells, LPS stimulation increased transcript levels of both cytokines, but NC-CV significantly decreased this LPS-induced upregulation (Figure 3A,B). We then assessed intracellular ROS production in BEAS-2B cells incubated with NC-CV, LPS, and both. Alone, NC-CV did not alter basal fluorescence compared with that of the untreated control cells (Figure 3C). On the other hand, while LPS markedly elevated intracellular ROS, co-treatment with NC-CV reduced this increase in a dose-dependent manner (Figure 3D). These results indicated that NC-CV can mitigate both the inflammatory response and oxidative stress caused by LPS stimulation in BEAS-2B cells.

### 3.3. NC-CV Treatment Suppresses LPS-Induced MAPK/NFκB Pathway Activation in BEAS-2B Cells

**Figure 4:**
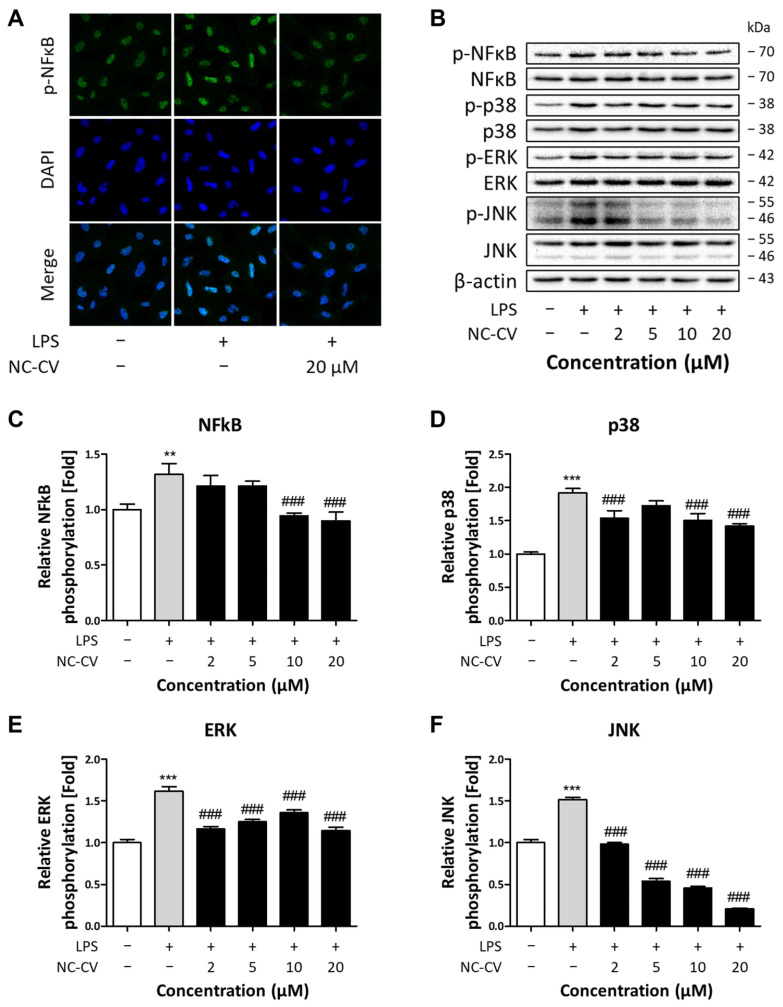
NC-CV suppresses LPS-induced activation of the NFκB and MAPK pathways in BEAS-2B cells. (**A**) Immunofluorescence images assessing the nuclear translocation of phosphorylated NFκB (green). Nuclei were counterstained with DAPI (blue). (**B**) Representative Western blot results showing the total levels of NFκB, p38, ERK1/2, and JNK as well as the levels of the four proteins’ phosphorylated forms. (**C**–**F**) The phosphorylated/total protein level ratios for each target protein, based on Western blot membrane band intensities. The data were expressed relative to the protein levels in the untreated control. Statistical significance is denoted using symbols: ** (*p* < 0.01) and *** (*p* < 0.001) for comparisons between the treatment and control groups and ### (*p* < 0.001) for comparisons between the NC-CV-treated and LPS only treated groups.

Lipopolysaccharide binds to TLR4 and rapidly activates MAPK family members, including ERK, JNK, and p38, as well as NFκB, thereby driving inflammation and ROS production [13]. To elucidate the mechanisms by which NC-CV exerts its anti-inflammatory effects, we first examined the nuclear translocation of NFκB, a key process that initiates pro-inflammatory gene expression. Immunofluorescence staining revealed that NC-CV suppressed LPS-induced nuclear translocation of phosphorylated NFκB (Figure 4A).

Since MAPKs are major upstream regulators of NFκB activation, we next evaluated the total and phosphorylated protein levels of MAPKs and NFκB in LPS-stimulated BEAS-2B cells using Western blot analyses. At 10 μg/mL, LPS significantly increased the phosphorylation of NFκB and all three MAPKs, whereas co-treatment with NC-CV markedly attenuated the LPS-induced phosphorylation in all cases (Figure 4B–F). Notably, NC-CV inhibited JNK phosphorylation in a dose-dependent manner, reducing it to levels to below those of the unstimulated control cells. These results demonstrated that NC-CV inhibits LPS-induced activation of both MAPK and NFκB signaling pathways in BEAS-2B cells.

### 3.4. NC-CV Exerts Antioxidative Activity Through Direct Radical Scavenging

**Figure 5:**
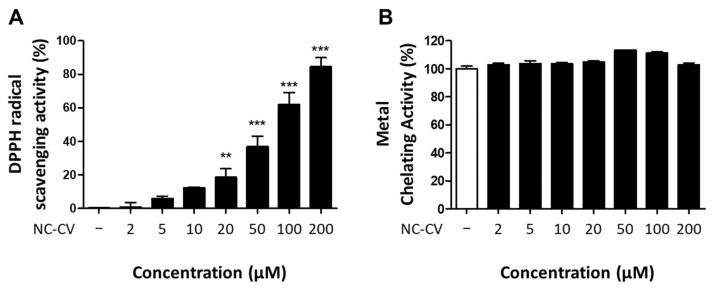
In radical-scavenging and metal chelating activity assays, NC-CV exhibited antioxidant capacity. To evaluate the direct antioxidant properties of NC-CV, two assays were performed: (**A**) DPPH radical scavenging activity is shown as the percent scavenging relative to that of the vehicle control. (**B**) Ferrous ion (Fe^2+^) chelation activity is shown as percent inhibition relative to that in the Fe^2+^-only control. Statistical significance for comparisons between the treatment and control groups is denoted using asterisks: ** (*p* < 0.01) and *** (*p* < 0.001).

As NC-CV reduced LPS-induced intracellular ROS stimulation, we investigated both direct and indirect antioxidant mechanisms to further elucidate its bioactivity. Mechanisms of direct antioxidant activity in functional peptides include radical scavenging and metal chelation [46,47]. To assess the intrinsic antioxidant activity of NC-CV, i.e., that independent of cellular signaling pathways, we performed DPPH radical scavenging and Fe^2+^ chelation assays (Figure 5A,B). Across a wide range of tested concentrations, NC-CV exhibited dose-dependent DPPH radical scavenging activity, whereas no Fe^2+^ chelation activity was detected at any concentration.

To further investigate the structural basis of this radical-scavenging activity, we compared NC-CV with other 18- to 20-mer peptides of varying amino acid compositions (Table 4). The compared peptides contained amino acids with known antioxidant properties, including tyrosine (Y) and tryptophan (W), which possess electron-rich aromatic rings; cysteine (C) and methionine (M), which contain electron-donating sulfur atoms; and histidine (H), lysine (K), and arginine (R), which contain electron-donating nitrogen atoms. Among all the tested peptides, NC-CV, which contains 13 amino acids with antioxidant potential, exhibited the highest radical scavenging activity, 84.58%. Despite having a greater number of antioxidant amino acids, Pep2, a 20-mer peptide with 14 antioxidant residues, exhibited only 52.03% radical scavenging activity. These results suggested that the specific composition and sequential arrangement of antioxidant residues within NC-CV, rather than their absolute number, contribute to its superior radical scavenging efficiency.

### 3.5. Effect of NC-CV on NRF2 and Antioxidant Enzymes

**Figure 6:**
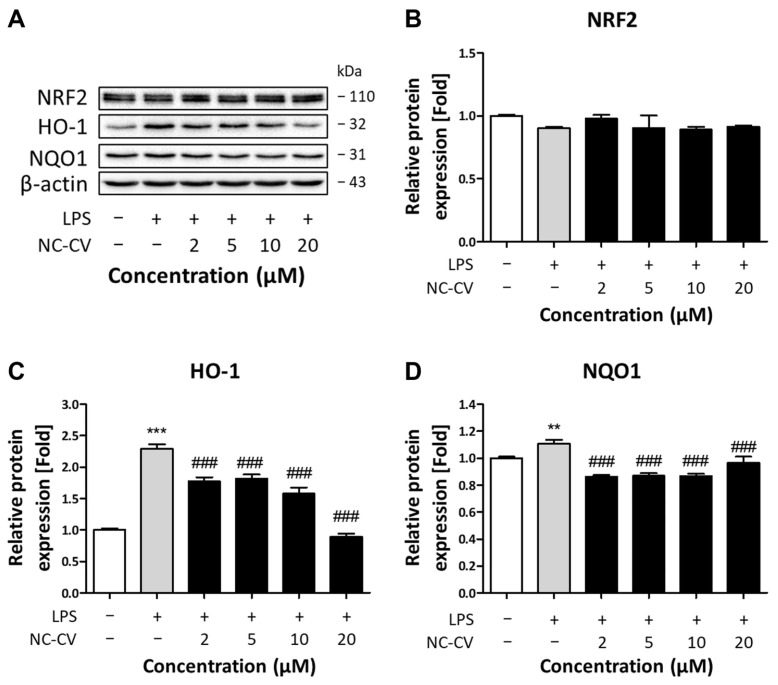
Effects of NC-CV on the NRF2 pathway-associated proteins in LPS-treated BEAS-2B cells. (**A**) Representative Western blot results showing total NRF2, HO-1, and NQO1 levels in BEAS-2B cells treated for 24 h with 10 μg/mL of LPS with or without 2, 5, 10, or 20 μM NC-CV co-treatments. (**B**–**D**) Relative protein levels of NRF2, HO-1, and NQO1 are expressed relative to the protein levels in the control. Statistical significance is denoted using symbols: ** (*p* < 0.01) and *** (*p* < 0.001) for comparisons between the treatment and control groups and ### (*p* < 0.001) for comparisons between the NC-CV-treated and LPS only treated groups.

The transcription factor NRF2 serves as a master regulator of cellular redox homeostasis, protecting cells against oxidative stress [48]. To investigate whether the antioxidant activity of NC-CV involves NRF2 signaling, we examined the protein expression levels of NRF2 and its downstream antioxidant enzymes, HO-1 and NQO1, in LPS-stimulated BEAS-2B cells. Neither the LPS treatment alone nor any LPS–NC-CV co-treatments altered total NRF2 protein levels (Figure 6A,B). However, NC-CV co-treatments attenuated the LPS-induced upregulation of HO-1 and NQO1, restoring them to basal levels at 20 μM NC-CV (Figure 6C,D).

**Figure 7:**
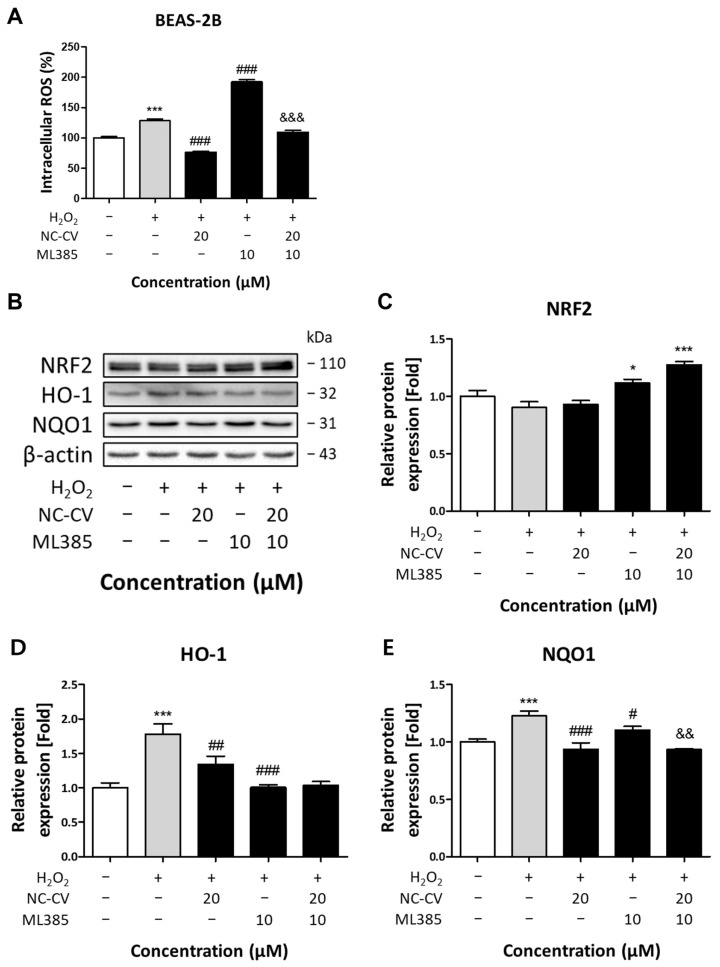
NC-CV exerts an Nrf2-independent antioxidant effect under non-inflammatory oxidative stress. (**A**) Intracellular ROS levels were measured using DCF-DA fluorescence in BEAS-2B cells treated with 75 μM H_2_O_2_ alone or in combination with 20 μM NC-CV, 10 μM ML385, or both. (**B**) Representative Western blots showing total Nrf2, HO-1, and NQO1 levels. Quantification of Nrf2 (**C**), HO-1 (**D**), and NQO1 (**E**), normalized to β-actin and expressed relative to the control. Statistical significance is denoted using symbols: * (*p* < 0.05) and *** (*p* < 0.001) for comparisons between the treatment and control groups, # (*p* < 0.05), ## (*p* < 0.01), and ### (*p* < 0.001) for comparisons between the NC-CV-treated and H_2_O_2_ only treated groups, && (*p* < 0.01) and &&& (*p* < 0.001) for comparisons between the NC-CV + ML385 + H_2_O_2_-treated and ML385 + H_2_O_2_-treated groups.

To further elucidate the antioxidant mechanism of NC-CV under direct oxidative stress conditions, we treated BEAS-2B cells with hydrogen peroxide (H_2_O_2_), which induces intracellular ROS independently of inflammatory signaling. Treatment with 75 μM H_2_O_2_ markedly increased intracellular ROS, and NC-CV completely negated this increase (Figure 7A). To determine whether this effect was NRF2-dependent, cells were co-treated with 10 μM of NRF2 inhibitor ML385. The ML385 treatment significantly increased intracellular ROS levels compared with those seen when applying the H_2_O_2_ treatment alone, confirming NRF2 inhibition; however, NC-CV addition still negated the H_2_O_2_-induced increase in intracellular ROS levels in the presence of ML385. Western blot analysis revealed that NRF2 expression was greater in ML385-treated cells than in those treated with H_2_O_2_ alone, both with and without the NC-CV co-treatment, while the expression of downstream enzymes HO-1 and NQO1 decreased in a similar fashion, confirming effective NRF2 pathway inhibition (Figure 7B–E). Collectively, these findings demonstrate that NC-CV exhibits potent antioxidant activity through direct radical scavenging that is independent of NRF2 downstream signaling under both inflammatory and non-inflammatory oxidative stress conditions.

### 3.6. Inhibition of TLR4 Dimerization by NC-CV Leads to Anti-Inflammatory and Antioxidative Effects

**Figure 8:**
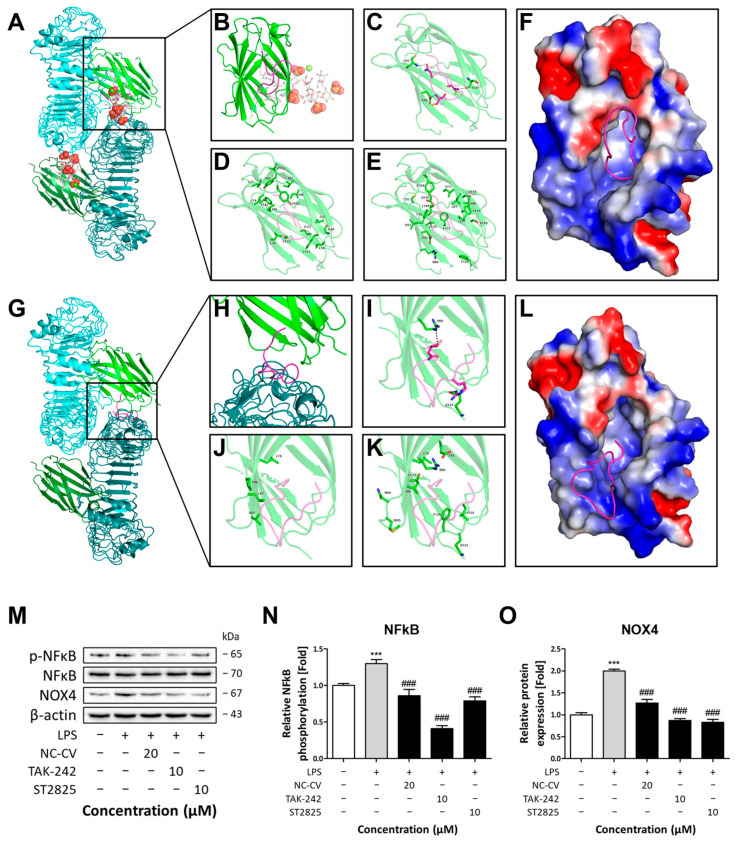
Molecular docking model of NC-CV–MD-2 binding. TLR4 and MD-2 are shown in cyan and light green, while the corresponding proteins of the complex are depicted in forest green and deep teal, respectively. The NC-CV peptide is represented as magenta cartoons and sticks. The interacting residues of MD-2 and NC-CV are labeled in black and gray, respectively. (**A**,**B**) Superimposition of the docking analysis results at the LPS binding site on the TLR4–MD-2–LPS complex. LPS is represented as white sticks. (**C**) Hydrogen bonding, (**D**) hydrophobic contacts, and (**E**) van der Waals interactions at the LPS binding sites on MD-2. Dotted lines represent interactions. (**F**) An electrostatic surface diagram of MD-2 showing positively charged surfaces as blue regions and negatively charged surfaces as red regions. (**G**,**H**) Superimposition of docking results at the dimerization interface of the TLR4–MD-2–LPS Complex. (**I**) Hydrogen bonding, (**J**) hydrophobic contacts, and (**K**) van der Waals interactions at the dimerization interface. Dotted lines represent interactions. (**L**) An electrostatic surface diagram of MD-2 shows positively charged surfaces as blue regions and negatively charged surfaces as red regions. (**M**) Representative Western blot results showing the protein levels of NFκB, phosphorylated NFκB, and NOX4. (**N**) The phosphorylated/total protein ratio for NFκB and (**O**) protein level of NOX4 normalized to β-actin. The data are expressed relative to the values seen in control-group cells. Statistical significance is denoted using symbols: *** (*p* < 0.001) for comparisons between the treatment and control groups and ### (*p* < 0.001) for comparisons between the pathway inhibitor (including NC-CV)-treated and LPS only treated groups.

To elucidate the mechanism by which NC-CV interferes with TLR4 signaling, we performed a molecular docking analysis to examine its interactions with the TLR4–MD-2–LPS complex. This analysis revealed that NC-CV competitively occupies the LPS-binding site of MD-2, a co-receptor crucial for TLR4-mediated LPS recognition (Figure 8A,B,F). This binding reaction is primarily mediated by van der Waals interactions and further stabilized by multiple hydrogen bonds and hydrophobic contacts (Figure 8C–E). Importantly, NC-CV interacts with S120, a residue critical to LPS-mediated TLR4 interactions, and also engages F126, a key residue for TLR4 complex formation (Figure 8D,E) [39,49]. Furthermore, NC-CV binds to the dimerization interface of the TLR4–MD-2–LPS complex, engaging residues V82, M85, and L87, which are essential for complex assembly (Figure 8G–L) [39]. This interaction is also predominantly driven by van der Waals forces with additional stabilization from hydrogen bonds and hydrophobic contacts (Figure 8I–K).

To experimentally validate the inhibitory activity of NC-CV on TLR4 signaling, we compared its effect with those of TAK-242, a TLR4 inhibitor, and ST2825, a MyD88 inhibitor. In BEAS-2B cells, LPS increased NFκB phosphorylation and the expression of NOX4, a TLR4-induced protein that mediates ROS production. Co-treatment with 20 μM NC-CV reduced LPS-induced NFκB phosphorylation to levels similar to those seen in control cells and comparable to those seen in ST2825-treated cells (Figure 8M,N). The NC-CV treatment also significantly reduced LPS-induced increases in NOX4 expression, though to slightly less of an extent than did the TAK-242 and ST2825 treatments (Figure 8M,O). Collectively, these results demonstrate that NC-CV attenuates NFκB phosphorylation and NOX4 upregulation, two major downstream effectors of TLR4 signaling that mediate inflammatory responses and ROS production, respectively. These findings are consistent with the TLR4–MD-2 complex formation interference computationally predicted via molecular docking.

## 4. Discussion

Respiratory diseases are driven by the interplay between persistent airway inflammation and oxidative stress, where repeated exposure to pathogenic or damaging signals amplifies epithelial injury and sustains disease progression [4]. Therapeutic approaches that simultaneously suppress inflammation and reduce oxidative stress in the airway epithelium are therefore highly desirable. Although corticosteroids remain the cornerstone of anti-inflammatory therapy, their long-term use is limited by significant adverse effects and incomplete efficacy, particularly in chronic diseases with steroid-resistant phenotypes [6]. These limitations highlight the need for safer, more effective alternatives suitable for long-term disease management. To address this challenge, we aimed to identify a novel functional peptide with dual anti-inflammatory and antioxidant properties through a spider venom gland transcriptome analysis combined with in silico methods.

Lipopolysaccharide, a component of the Gram-negative bacterial outer membrane, is recognized by the TLR4–MD-2 complex, which activates NFκB and MAPK signaling, leading to the release of pro-inflammatory cytokines that drive airway inflammation and tissue injury [9,12]. This inflammatory response is coupled with excessive ROS generation via NADPH oxidases and mitochondria, which overwhelms cellular antioxidant defenses, damages cellular macromolecules, and triggers respiratory epithelial cell death [13,14]. These interconnected processes form a pathogenic cycle that sustains pulmonary inflammation and oxidative stress [50]. Therefore, we used an LPS-stimulated BEAS-2B cell model to evaluate the therapeutic potential of NC-CV. We showed that NC-CV mitigated LPS-induced adverse effects by improving cell viability, reducing intracellular ROS and downregulating pro-inflammatory cytokine expression. Such simultaneous inhibition of both inflammatory and oxidative stress suggests that NC-CV may aid in preserving epithelial integrity and disrupting the pathogenic cycle characteristic of LPS-induced airway injury.

Mechanistically, we showed that NC-CV significantly suppresses the phosphorylation of NFκB and MAPK-pathway proteins in LPS-stimulated BEAS-2B cells, leading to a reduction in the inflammatory response. This biological effect is supported by the molecular docking analysis, which revealed that NC-CV competitively binds to key sites within the TLR4–MD-2–LPS signaling complex. Specifically, it occupies the LPS-binding pocket of MD-2 and interacts with critical residues required for complex formation and receptor activation, including F126 and S120. These interactions are strengthened by multiple hydrogen bonds and hydrophobic contacts, which help stabilize NC-CV binding. Additionally, NC-CV engages the dimerization interface, namely residues V82, M85, and L87, suggesting another mechanism by which it may inhibit complex assembly. By simultaneously targeting the LPS-binding pocket and the dimerization interface, NC-CV is able to block TLR4–MD-2–LPS dimeric complex formation, preventing proper receptor association and downstream signal transduction. Functionally, this interference mirrors the effects observed with established TLR4 and MyD88 pathway inhibitors, such as TAK-242 and ST2825, resulting in marked suppression of TLR4-dependent signaling. Our molecular docking analysis, pharmacological pathway inhibition studies, and downstream signaling assays collectively support the TLR4–MD-2 targeting mechanism. However, definitive validation will require TLR4 loss-of-function models (siRNA, CRISPR editing, or TLR4-deficient cells), which we will address in future work. Consequently, through reductions in both LPS recognition and TLR4 activation, NC-CV may alleviate cellular stress triggered by TLR4 agonists, not only by dampening the initial inflammatory response but also by reducing subsequent oxidative stress mediated by NOX activation and cellular damage. Overall, these findings provide mechanistic insight into how NC-CV acts as an inhibitor of TLR4-mediated signaling, underscoring its potential as a modulator of innate immune responses.

Antioxidant peptides typically act through direct radical scavenging, the chelation of pro-oxidant metal ions, or the enhancement of endogenous antioxidant defenses [47,51]. Among these mechanisms, NC-CV was shown to primarily act as a direct radical scavenger, rather than by modulating endogenous antioxidant pathways. In both LPS- and H_2_O_2_-induced oxidative stress conditions, NC-CV significantly reduced the elevated intracellular ROS levels, and it showed dose-dependent radical scavenging activity in DPPH assays. Examination of the NRF2 pathway revealed that co-treatment with NC-CV decreased the expression of NRF2 downstream targets HO-1 and NQO1 in LPS-stimulated cells, suggesting that NC-CV’s antioxidant effects lessen oxidative burden and decreases the need for compensatory enzyme induction.

To further confirm this NRF2-independent mechanism, we examined NC-CV’s antioxidant activity in the presence of the NRF2 inhibitor ML385. Both ML385 treatment alone and the co-treatment with ML385 and NC-CV resulted in significant increases in NRF2 expression. Under ML385 treatment, elevated ROS levels can lead to weakened KEAP1-NRF2 binding, contributing to NRF2 accumulation in the cytoplasm, while HO-1 and NQO1 expression remains significantly suppressed due to the ML385-mediated inhibition of NRF2 transcription. Interestingly, in the ML385–NC-CV co-treatment group, NRF2 accumulation persisted despite reduced ROS levels, and NQO1 expression was suppressed to an even greater extent than that seen with ML385 alone. This suggests that NC-CV effectively suppressed H_2_O_2_-induced ROS regardless of NRF2 pathway activation, where it may also regulate NRF2 downstream targets through mechanisms beyond simple ROS reduction.

The efficiency of antioxidant peptides in eliminating ROS is determined not only by the number of antioxidant-related amino acids, but also by their specific composition and arrangement. Compared to other antioxidant-related peptides examined in the study, NC-CV exhibited superior radical scavenging activity. For example, despite having more antioxidant amino acids, Pep2 showed about 28% lower antioxidant capacity than NC-CV, while Pep3, with 11 residues, had the lowest activity among those tested. These findings highlight the fact that the spatial arrangement and context of antioxidant residues are critical for radical scavenging efficiency, suggesting that NC-CV possesses a structurally optimized configuration for direct ROS scavenging.

This study provides an in vitro mechanistic proof-of-concept focused on identifying NC-CV and characterizing its dual anti-inflammatory and antioxidant activities. Therapeutic peptides require adequate bioavailability and systemic exposure to achieve efficacy while maintaining low cytotoxicity. Therefore, it would be beneficial to determine proteolytic stability in human serum, hemolytic potential, and in vivo half-life, as well as tissue-distribution kinetics in appropriate animal models. To further improve pharmacokinetic properties, peptide sequence-level optimization strategies could be considered, such as cyclization, N- and C-terminal capping, and incorporation of non-natural amino acids, to increase protease resistance while maintaining biological activity [52,53,54]. Such optimization may also enhance stability and cellular uptake, creating a more advantageous profile for therapeutic development. Additionally, this study showed that NC-CV attenuates cell-surface TLR4–MD-2 signaling, which suppressed NFκB/MAPK activation and NOX4 expression under LPS stimulation, and significantly reduces intracellular ROS in BEAS-2B cells. Under H_2_O_2_-induced conditions, intracellular ROS was also decreased, indicating antioxidant effects even in the absence of receptor-dependent signaling. These findings suggested that NC-CV exerts antioxidant effects through both inhibition of the extracellular receptor and direct radical scavenging following intracellular translocation. Several studies have demonstrated that cationic, α-helical peptides can be internalized via endocytosis or direct membrane translocation; electrostatic binding to heparan sulfate proteoglycans and alignment of amphipathic α-helices with the phospholipid bilayer [55,56]. In the case of NC-CV, the physicochemical features are consistent with these properties, supporting the possibility of internalization by its intrinsic properties.

Collectively, this study demonstrates that NC-CV attenuates airway inflammation and oxidative stress through dual mechanisms: the disruption of LPS-induced TLR4–MD-2 complex formation and the direct scavenging of free radicals, independent of NRF2 signaling. These effects were validated by demonstrating its ability to inhibit NFκB/MAPK phosphorylation, suppress pro-inflammatory cytokine production, and significantly reduce intracellular ROS. Given these promising in vitro findings, further evaluation of NC-CV in vivo using respiratory disease models will be essential to elucidate its therapeutic potential within the complex interplay between airway tissue and immune cells. Concurrently, optimization strategies to enhance peptide stability and bioavailability—such as the incorporation of non-natural amino acids or cyclization—will be critical for advancing NC-CV toward clinical application. In summary, the mechanistic insights obtained in this study suggest that NC-CV is a promising therapeutic candidate for simultaneously targeting the interconnected inflammatory and oxidative pathways that drive respiratory disease pathogenesis. Notably, unlike TLR4-directed peptides that act primarily as receptor antagonists without intrinsic antioxidant capacity, NC-CV showed receptor-level interference at the TLR4–MD-2 axis with direct radical scavenging under the same experimental conditions. This dual profile distinguishes NC-CV as a single agent that attenuates pro-inflammatory signaling while concurrently lowering the oxidative burden in the airway epithelium.

## 5. Conclusions

This study demonstrates that the spider venom-derived peptide NC-CV exerts potent dual anti-inflammatory and antioxidant effects in airway epithelial cells under LPS stimulation. By competitively binding to the TLR4–MD-2 complex, blocking LPS recognition and inhibiting NFκB/MAPK signaling, NC-CV suppresses pro-inflammatory cytokine release. Additionally, NC-CV acts as a direct radical scavenger, effectively reducing intracellular ROS levels independently of NRF2 activation. These mechanistic insights demonstrate NC-CV’s unique ability to disrupt the cycle of inflammation and oxidative stress that underlies respiratory epithelial injury. Taken together, our findings position NC-CV as a promising therapeutic candidate and showcase the potential of venom-derived peptides in the development of novel treatments for inflammatory airway diseases.

## Figures and Tables

**Table 1:** Target genes and sequences of the primers used in the study.

Target Gene	Forward Primer (5′ → 3′)	Reverse Primer (5′ → 3′)
β-actin	AACTGGAACGGTGAAGGT	CCTGTAACAACGCATCTCATAT
IL-1β	AGGATATGGAGCAACAAGT	ATCATCTTT CAACACGCAG
TNF-α	TTAAGCAACAAGACCACCA	CTCCAGATTCCAGATGTCA

**Table 2:** Physicochemical properties of NC-CV, the selected peptide derived from *Nephila clavata*.

Peptide	Sequence	Molecular Weight	Net Charge	Water Solubility	PI
NC-CV	CVNHCTRHRHSCCRSKMK	2186.56 g/mol	+5	Good	10.22

**Table 3:** Machine learning-based functional prediction results for the NC-CV peptide.

Peptide	AIP Prediction			AOP Prediction	Hemolysis Prediction	
	PepNet	PreAIP	AIPID	AnOxPpred	Peptide.bio	DBAASP
NC-CV	0.713	0.653	0.81	0.482	Not active	Not active

**Table 4:** Radical scavenging activities and antioxidative amino acid compositions of the tested peptides.

Peptide	Sequence (Length)	Net Charge	Antioxidative Amino Acids (No. of Residues)				Radical Scavenging Activity (%)
			Y, W	C, M	H, K, R	Total	
Pep1	VVSTTSYCKKMKKDCNDYTK (20)	+2.9	2	2	5	9	72.57
Pep2	VRKLTRYCKKMKKDCKRYWK (20)	+8.9	4	2	8	14	52.03
Pep3	GADCCVVSTTSYCKKMKKDC (20)	+1.7	1	3	4	8	59.44
Pep4	AAKRCVRSWTRYCKKMKKDC (20)	+6.8	2	2	7	11	28.75
Pep5	MMGHRCRAMKCMKKVHDK (20)	+5.1	0	5	7	12	57.50
Pep6	MMKHMCRAMKCMKKVMDK (20)	+5	0	6	6	12	69.75
Pep7	TCVNHCTRHRHSCCRSKM (18)	+4	0	5	7	12	63.14
NC-CV	CVNHCTRHRHSCCRSKMK (18)	+5	0	5	8	13	84.58

## Data Availability

The original contributions presented in this study are included in the article/Supplementary Materials. Further inquiries can be directed to the corresponding author.

## Supplementary Materials

The following supporting information can be downloaded at: https://www.mdpi.com/article/10.3390/antiox14121485/s1. Table S1: Homology, signal peptide cleavage position, and TPM values of 37 putative toxin/venom transcripts; Table S2. Mature protein sequences of toxin/venom transcripts derived from the *N. clavata* transcriptome; Table S3. Comparison of dimerization affinities between native complexes and NC-CV–bound interfaces; Figure S1: Quality control statistics for the synthesized NC-CV peptide; Figure S2. Cell viability test on the THP-1 and HDFa cell.
