# Supplementary material for: Spider Venom-Derived Peptide Exhibits Dual Anti-Inflammatory and Antioxidative Activities in LPS-Stimulated BEAS-2B Cells
Source: Antioxidants (Basel). 2025 Dec 11;14(12):1485. doi: 10.3390/antiox14121485 (PMC12729936; doi:10.3390/antiox14121485)
Supplement: Supplementary file 1 [file antioxidants-14-01485-s001.zip › antioxidants-3986812--supplementary.pdf]

Supplementary materials

Spider Venom-Derived Peptide Exhibits Dual Anti-Inflammatory and Antioxidative Activities in LPS-Stimulated BEAS-2B Cells

Table S1. Homology, signal peptide cleavage position, and TPM values of 37 putative toxin/venom transcripts.

Query Sequence ID	E-value	Query coverage	SpiderP cleavage Site position	Female TPM average	Male TPM average
DN6582	7.03E−12	71	21	582.9613737	289.4140687
DN117376	0.001	95	19	244.6139353	193.5610717
DN449	3.49E−14	84	17	297.7133773	5.888072333
DN3583	5.29E−30	67	20	62.18704067	11.889353
DN364	2.35E−06	85	20	10.33083233	127.7578337
DN4934	2.34E−65	99	22	4414.525727	30.87103267
DN93804	5.18E−06	64	24	82.461357	9.062988667
DN6764	2.79E−06	72	19	100.3162523	40.68157633
DN4171	1.90E−28	93	27	74.50034833	76.17701567
DN6919	9.41E−25	99	18	404.383024	214.3229763
DN10603	3.84E−15	74	20	95.76800633	78.258985
DN364	1.58E−12	94	22	20.02545067	506.5178313
DN4934	4.95E−07	83	32	95.48528633	99.60572567
DN93804	8.33E−07	63	19	41.33265633	136.3226423
DN6764	6.28E−58	95	22	18840.9137	219.764432
DN4171	8.40E−51	91	20	65.64616167	44.05364567
DN6919	0.044	70	19	37627.47036	435.6503217
DN10603	1.89E−08	94	23	0.960903333	4323.929061
DN2785	0.000438	68	42	0	51.27105233
DN376	0.016	89	19	169.886296	44.87145933

DN4617	5.05E−31	91	18	372.1877377	307.08265
DN38_c0_g1_i8	0.000108	73	21	0	1380.888487
DN9475	1.00E−06	82	21	0.036134	1586.181406
DN178	0.002	77	21	0	981.117097
DN38_c2_g1_i5	5.48E−21	89	21	6.859881333	1607.719072
DN23425	3.36E−33	86	21	0.057093	8137.533019
DN93607	5.78E−42	99	19	154.700547	88.92087367
DN607	8.14E−12	63	15	71.206779	17.62912067
DN82_c0_g1_i5	1.36E−36	78	19	131.3273837	2.779489
DN82_c3_g2_i1	7.11E−44	96	21	262.651917	11.50346
DN1997_c1_g1_i4	1.28E−23	99	22	140.4605567	1.007866
DN82_c0_g1_i4	5.29E−11	96	22	144.0263547	22.42441767
DN82_c0_g1_i3	6.54E−11	96	22	154.701096	8.654199333
DN7123	6.60E−20	95	22	86.59672567	1.414444667
DN397	0.012	81	20	28.35987533	110.027834
DN1942	0.036	72	16	5540.774601	451.759983
DN2254	5.75E−39	97	21	1.724844333	2885.355367
DN201	7.03E−12	71	21	582.9613737	289.4140687
DN214_c0_g1_i13	0.001	95	19	244.6139353	193.5610717
DN214_c0_g1_i4	3.49E−14	84	17	297.7133773	5.888072333
DN214_c0_g1_i7	5.29E−30	67	20	62.18704067	11.889353
DN13476	2.35E−06	85	20	10.33083233	127.7578337
DN1038	2.34E−65	99	22	4414.525727	30.87103267
DN1327	5.18E−06	64	24	82.461357	9.062988667

Table S2. Mature protein sequences of toxin/venom transcripts derived from the *N. clavata* transcriptome.

Query Sequence ID	Mature sequence
DN6582	AANHAAGSKSGKHHKAKVKAKRVVAGTNYMDVGMTCKKDVDAGCGVSTYKKCTVVKDNGKHKVNTGCASKKD
DN117376	ADKCGDCCVGNTVGMCRKRHVDVCMKMKNKDHVYKRCCNGKCVSSKGGGKAGKCDSGS
DN449	ARRMADNWMKCNKNGTACSDYDVDTCMCCTDGTTWGNKSCVCAYHDHDAKCNKDGSYACSTKCWCVDRNGTVVSKDVHSCD
DN3583	ASNDDVDYYGRATDDCCVYCVDGVDDGSNDRKKTDKYVCGHVCCRNTSTRYHRCGTRNGGNGRSAHANGAGWWVAKNDNGKCGGTDDRHTAHCVDKDNKSMVVRGWDTNTDMHDYGVDMHYRSNNNDAVVRDRVVKHDTACDDDDTGCVATGWGTDAYGSMKTVSNSCNRKTRGRKYGGMCAGGKAGDACKGDGGGVCRSDNSYTVAGVSWGDCGGGVYVNVKKYNDWVSKTKNYWSSSR
DN364	CCVGMRYSSAWCKGKDDCRSDNAVDRYNGVKHHDVHCCNNCSRACTSARTNDVSRSS
DN4934	CSHHSYCSNSCAYYVNDKKVHHNMYRNVATGKTRAANMMWDAAVAKYADCRYHDSNCRRVMNGVGNARTGGHTVADWDAVNDWHVYSNDYSGSYRHSAWADSRVGCGYVYKGNTYTRYCNYGAGNVYGGNVYRGACSDCVNTCCGSSCTMTSHGCRRTSDNYMNYCANNTDCNNYMGNNWVTGGNYGVNGGNSTVTNKKSDTCVSMYRKGNKYDDMANADVSNYTVSGYNVRSTNTSWDKTMKSAVGKANVKKAVDGSCN
DN93804	DCCVARGGRKGNCKKAKKDACSKDVVADYGHCCVDGSCTVKDGTVKDRCVGGSTTATTAA
DN6764	DCGCCVNYVVSGGHCKKRGSCTKDDGKDMYHCCKGDCKAKHKGHKHRCVVGDDKD
DN4171	DGDARNAGYRWNARVYVDYSGGYSRMNAAYATTCVRVRTNRDYYGGCYSHVGKTGGSGNGCYGTVVHGHAGYHNRSDRDDHYNVVRGMSN
DN6919	DRARRNATTSHCDNGDYAACTSNDWCVCYRRNGNNTSKTKACDCRHDDAKTAGDTYVCRDGHYRSKCRDCWCVDKDGKVTDVGDVSC
DN10603	DVNSKYARMATKNTDSMHSHKMMNTKAKVVSGMNYKMVGTCRRNGNYTDDCKCDNVCTVVWVWRTNVSTRSSCAVGDTC
DN364	DWHGSWYDKKCCVYDTYGYGCGKGHKCWVGKGVNYTCCVCKNGW
DN4934	GCDSCVNARVSDGCDDKGVCCTTNADCRGGRCGTDTCSNVNGDCSGTRCCY
DN93804	GGGMMGHRCRAMKCMKKVHDKKNGSGSCRDANRNCKNMKARAKSSCVYGVSTTTTSTSSY

DN6764	HSYCNNCRDTVSDDKDDVVRAHNYRDKVATGRSAAGGMTAANMMMVWDDASAKHATCKSHDCYACRVDRVGNYMAMSWGYDKSYWRMVKAYDDKSYVGSYGHTVVWATSWRVGCGKAVKDKKWYKTYVCNYGAGNMMGGMYKGDTCSCSNTCCGSSCAYGSDYGCKVDDNSK
DN4171	HYSGYNATKGRHHYWVSRSAGDWNGGGCSSDGSGHVSADGKTYNNTYAWNRVANVAAGVGSYADDKKYTDDDSVSYDNYAHKDKNAYTGSYGGYTSVRVTGADNKGAGNGYDGTNTNSVAYYHGGWSSAYCCDGHASTCDAGDSKDCDASVTNVVNGNVYNYSDCAHSKKTSRYAVDKRNRYKKGMTDDCTDSSNRKWNSVRAHTHVDWDCSDVGYKRYDTMRKGSGKRGYNGDVDMACNGDWSYNGKVTKDRWTNGAGVKNYGKTMTKGSGHMVDKGAKMNSNK
DN6919	KDRYNARDSDAKGRARKCGACSASGNCCSRTRCVWDRGSDGKGNAKWTSHCRYNGGVWVDNNKNG
DN10603	KHAGMWNDNKCMCDDTYGYGCGKHKCTVVGRGKYDCCDCNGAW
DN2785	MGVDKAKARDRVCCYRSDVTSCRSNCKNDYMKCVDARKDKADYMVNHYGR
DN376	NDCDGCCVRMSKAKCRKKHCNSHGDGYHYMCCAGKCAKKGSKTYGGGNCRKV
DN4617	NNDSSNTRVTGGHNAGGRYASCDATWRARDTCGATTDSHVTAAHCVKNRYSDVVDGDYDDGGHRKARSVYRDHSHSDARVVWRSGVKAAWSGTVSVYGWGRRYDGGHKVSVVVDNCKSVTHKMCAGGKGHDSCGDSGSGVVRDNVCGVSGRRCAMHVGVYTRVSSYVWYKNTSSNCKCYDVATSNMDSASKSNR
DN38_c0_g1_i8	NRASSSNCVCGGNKTRVDSVNHKYWVVGVRKYYSDDYCGGASRHYVTAAYCVNTHNTRVVGAYDSRSNVS
DN9475	NTKRASSSSRSCCGRNVKTKRVGGSAVDHRYWVVAVRRTYNTDKYCGGASYVTAAHCTSTVNTRVAGAHDSTSSVRSMAHYTNDSYDGK
DN178	NVSNKNRSADKTHGVSYKCNMGCDCTGCCTGYYDRRSNRCRKVYK
DN38_c2_g1_i5	RASSSSNCRTCGRNTNTGRSGTVNHRYWMVAVNKRDMDDYCGGVSYYVTAAHCKDTHNTRVVGAYDSRSNVSAHDGRDYDGKTAAGNVNVACTSSDGRKKTVGWRMSMNDGRKVDANDYCRAGRHRDGTCATYTRDACANDSAGVNHDKWAGRDWSMKCGVTGTRVSKYWSTRDYDCTGYKMKNDNCGNMSRAGGSKAAWMAYNDGSGAVSKVTAASDWMKDHKTVGKHKSASHKRNVKVVHHRYNTHNNDAHKKAADSYRCRKDYNTVTAGWGDNHRSSHKADMKDVCKDKYWNKRMCVNVDACKGDGGAMRYGRYYAGVASWNSTGCRVKGRVSAVRSSWSNRAKDVV
DN23425	RNRSSRNCVCGNVRTKRSGGSAVNHRYWVVAVRRKHNRDDYCGGASRHYVTAAHCNTRNTRVAGAHDSSTSVSMDHYRDDSYDVGVKTAGNVNVCTDSDGDMATVAGWGYHTYGGKRVDRHSDRCSYHRTCAGGRGKSSCMGDSGGKDGKWYGVAVAWNADCGRGTYTRTYWRTRDSCVKTYDSCGNMSRAGGSKAAWMAYNGGSGASKVTAASVRDRMKNHTVTGKHGV

DN93607	RRKSNTKVKVKCASNGDYACGSCMCWRDGSHTDSKHKTCVCHAHRDKSKTKTGMVGNTCNDGTYARKCHGSTGYCWCADKGNKSDVRGGANC
DN607	SCGTACTCDNYKNRACVMCNGCHCDGYVKAKDGSCVVCSSAADCGVNRYTGCGTACTCDNYKNKCNMCMGCCDGYVRSRGRCVRCRSKNCHADGGMCRGYMWYDSDSTCKYGGCGNGNRYATKCRTCASVWDSDDSNVCDATGRCRGRYHDKSTGCKRVYGGCGGNGNNKTDCNCGNAASVVSACKVVGCRAAGRYNKTGCHYGGCGGNDNNSDDCAVC
DN82_c0_g1_i5	SDCDCCDSKHACRYRGRCTASYKDYACCVDKYCGKGSNGVTVKDAKCTV
DN82_c3_g2_i1	SNGVKMVNCDTNGDYMMCGSKCSCYDKSGNVTSTKKSCNCVKRYANKKGNCTDGTYKKRCNGSTGYCYCVNMTGKKGDAKRGMVNC
DN1997_c1_g1_i4	TTDKYRSACRHHDCTKRHNCCRSKMKDKCKCYAKNDTTACTCSWYYNMTKAKTKKHG
DN82_c0_g1_i4	VNNDKRKACKKHHCTHDKNCCGRKYKCKCYDVTNGRCACRNTKNKAANGKNAN
DN82_c0_g1_i3	VNNDKRKACKKHHCTHDRNCCGRYKCKCYDVTNGRCACRNTKNKAANGKNAN
DN7123	VNNDKRSTCVNHCTRHRHSCCRSKMKKCTCYKGNDTSTDDCTCVWYHAADDRGTKNWGG
DN397	WMCASGDDMCNAGCCVKDGTTKNSTKTCKCHRNVASRKMVGSYKNCDGNYKKVCSGSTGSCWCVDNGKRNNRTSCSK
DN1942	WVADMKVKKGGMADRNKCYGKKCSHSCCKGAACVDGSGTCHGKYCKDSDCGAGVCDSGDASKTCGRSKKG
DN2254	YGDYTNGSKSNDTKRNTRNGKWNGVYTDYSSKRSAMSKTCKWKTDDNYVYSMGCWSRYGRVGGVDSSRCMKGMHMMHTGHSRDRDYVVWDNDGMKNKSDMTDGDYDYRSTHYRAWMYANTTKNDSVGYGTGTDKNKYCS
DN201	AANHAAGSKSGKHHKAKVKAKRVVAGTNYMDVGMTCKKDVDAGCGVSTYKKCTVVKDNGKHKVNTGCASKKD
DN214_c0_g1_i13	ADKCGDCCVGNTVGMCRKRHVDVCMKMKNKDHVYKRCCNGKCVSSKGGGKAGKCDSGS
DN214_c0_g1_i4	ARRMADNWMKCNKNGTACSDYDVDTCMCCTDGTTWGNKSCVCAYHDHDAKCNKDGSYACSTKCWCVDRNGTVVSKDVHSCD
DN214_c0_g1_i7	ASNDDVDYYGRATDDCCVYCVDGVDDGSNDRKKTDKYVCGHVCCRNTSTRYHRCGTRNGGNGRSAHANGAGWWVAKNDNGKCGGTDDRHTAHCVDKDNKSMVVRGWDTNTDMHDYGVDMHYRSNNNDAVVRDRVVKHDTACDDDDTGCVATGWGTDAYGSMKTVSNSCNRKTRGRKYGGMCAGGKAGDACKGDGGGVCRSDNSYTVAGVSWGDCGGGVYVNVKKYNDWVSKTKNYWSSSR
DN13476	CCVGMRYSSAWCKGKDDCRSDNAVDRYNGVKHHDVHCCNNCSRACTSARTNDVSRSS

DN1038	CSHHSYCSNSCAYYVNDKKVHHNMYRNVATGKTRAANMMWDAAVAKYADCRYHDSNCRRVMNGVGNARTG<br>GHTVADWDAVNDWHVYSNDYSGSYRHSAWADSRVGCGYVYKGNTYTRYCNYGAGNVYGGNVYRGACSDCVN<br>TCCGSSCTMTSHGCRRTSDNYMNYCANNTDCNNYMGNNWVTGGNYGVNGGNSTVTNKKSDTCVSMYRKGNK<br>YDDMANADVSNYTVSGYNVRSTNTSWDKTMKSAVGKANVKKAVDGSCN
DN1327	DCCVARGGRKGNCKKAKKDACSKDVVADYGHCCVDGSCTVKDGTVKDRCVGGSTTATTAA

Table S3. Comparison of dimerization affinities between native complexes and NC-CV-bound interfaces

Target	Interface area*	Delta G (kcal/mol)**	Binding E (kcal/mol)***	*P*-value
MD2-LPS	245.0	−0.3	−0.3	0.0408
MD2-NC-CV	1219.0	−18.1	−20.3	0.1556
MD2-TLR4	498.8	−7.1	−8.8	0.1187
MD2-NC-CV	681.4	−11.8	−13.1	0.1438

* Interface area = (SASA_A + SASA_B − SASA_AB)/2)

** Binding free energy (ΔG = ΔG_solvation + ΔG_interface + ΔG_entropy)

*** Binding energy (BE = E_solvation + E_specific interactions)

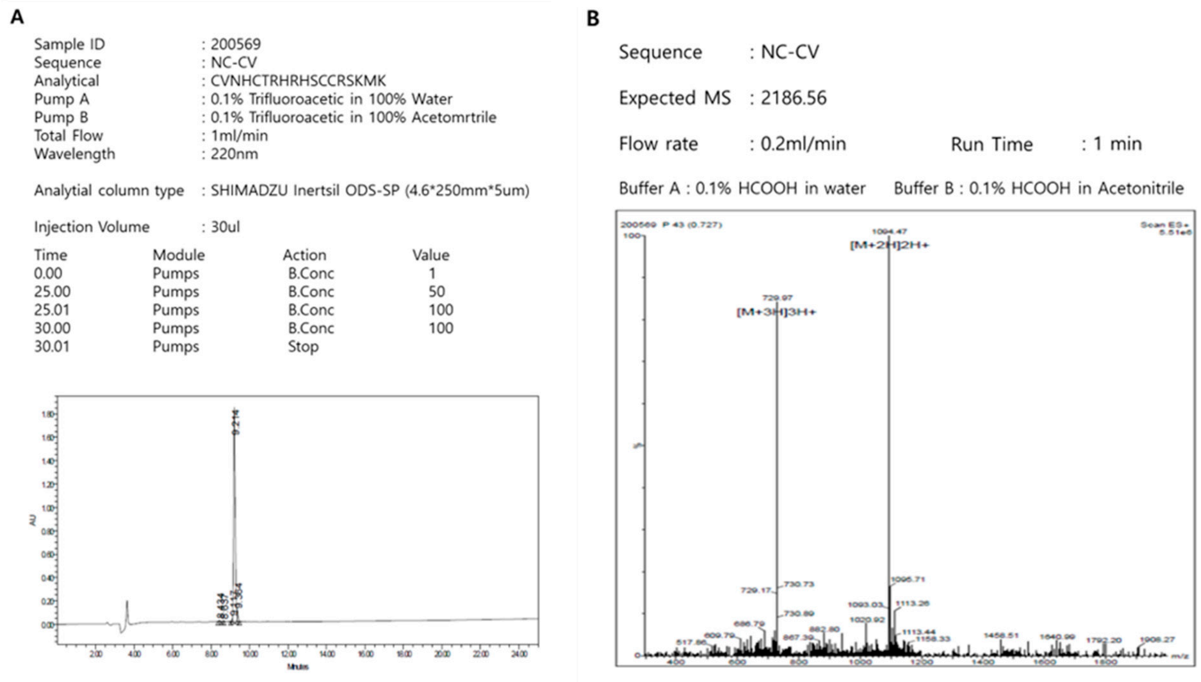

Figure S1. Quality control data for the synthesized NC-CV peptide.

(A) HPLC profile and (B) MS chromatogram of the NC-CV peptide were presented. The peptide was collected with a purity >95.20%.

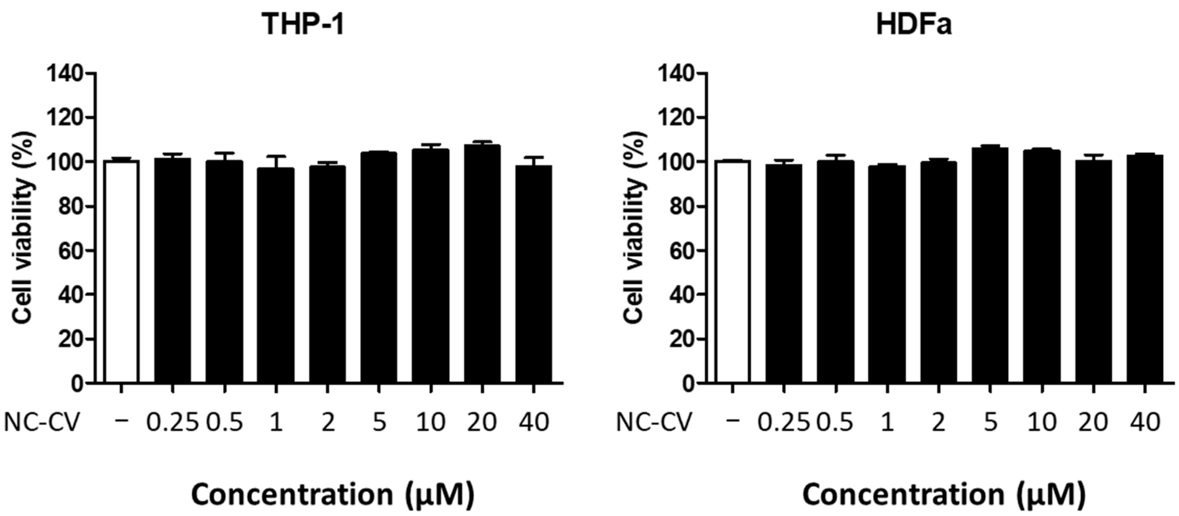

Figure S2. Cell viability test on the THP-1 and HDFa cells.

To evaluate cell cytotoxicity of NC-CV beyond epithelial cells, cytotoxicity was further assessed in (A) human macrophage-like cells (THP-1) and (B) human dermal fibroblasts (HDFa).
